# Osteopathic manipulative treatment showed reduction of length of stay and costs in preterm infants: A systematic review and meta-analysis

**DOI:** 10.1097/MD.0000000000006408

**Published:** 2017-03-24

**Authors:** Diego Lanaro, Nuria Ruffini, Andrea Manzotti, Gianluca Lista

**Affiliations:** a Clinical-based Human Research Department, C.O.ME. Collaboration; b SOMA—School of Osteopathic Manipulation, Milano; c NICU-“V.Buzzi”-Ospedale dei Bambini-ASST-FBF-Sacco-Milan-Italy.

**Keywords:** length of stay, NICU, osteopathic manipulative treatment, preterm infants, systematic review

## Abstract

**Background:**

Osteopathic medicine is an emerging and complementary method used in neonatology.

**Methods:**

Outcomes were the mean difference in length of stay (LOS) and costs between osteopathy and alternative treatment group. A comprehensive literature search of (quasi)- randomized controlled trials (RCTs), was conducted from journal inception to May, 2015. Eligible studies must have treated preterm infants directly in the crib or bed and Osteopathic Manipulative Treatment (OMT) must have been performed by osteopaths. A rigorous Cochrane-like method was used for study screening and selection, risk of bias assessment and data reporting. Fixed effect meta-analysis was performed to synthesize data.

**Results:**

5 trials enrolling 1306 infants met our inclusion criteria. Although the heterogeneity was moderate (*I*^2^ = 61%, *P* = 0.03), meta-analysis of all five studies showed that preterm infants treated with OMT had a significant reduction of LOS by 2.71 days (95% CI −3.99, −1.43; *P* < 0.001). Considering costs, meta-analysis showed reduction in the OMT group (−1,545.66€, −1,888.03€, −1,203.29€, *P* < 0.0001). All studies reported no adverse events associated to OMT. Subgroup analysis showed that the benefit of OMT is inversely associated to gestational age.

**Conclusions:**

The present systematic review showed the clinical effectiveness of OMT on the reduction of LOS and costs in a large population of preterm infants.

## 1 Introduction

Prematurity is a serious health care problem.^[1]^ Compared to full-term newborns, the likelihood of being affected by poor health, developmental and cognitive delays within the first year of life is higher in premature babies.^[2,3]^ This in turn will result in extensive psychological, physical, and economic costs.^[1–3]^ One of the main factor contributing to costs is length of hospital stay (LOS),^[4]^ also considered a proxy of health infant status.^[4–7]^

Osteopathic medicine is a noninvasive, drug-free manual medicine, classified as a complementary and alternative medicine and works through manual manipulation techniques^[8–10]^, which has been shown to be 1 emerging strategy to improve newborns’ health outcomes. One of its core concepts is somatic dysfunction (SD) defined as “impaired or altered function of related components of the somatic (body framework) system: skeletal, arthrodial and myofascial structures, and their related vascular, lymphatic, and neural elements.”^[11]^ SD is diagnosed on the basis of specific palpation criteria, such as tissue alteration, asymmetry, range of motion and tenderness, also known as TART.^[11]^ The cranial strain pattern, consisting in a somatic dysfunction of the head, is defined as a membranous articular strain due to abnormal dural tension. The 2 core components of osteopathic health care are the patient structural diagnostic evaluation, followed by the application of range of manual manipulative treatment techniques.^[28]^. A specific osteopathic evaluation in newborns has been described in the literature.^[12]^ Studies seem to report possible correlation between SD and specific clinical conditions, although more robust data are needed.^[13,14]^

Specific metabolic and neurological alterations were identified in SD *situ.* Those include hypersympathetic state^[15]^ and metabolic changes.^[16,17]^ Generally, in-vitro and in-vivo research suggested that osteopathic manipulative treatment (OMT) has anti-inflammatory and parasympathetic effects.^[18–21]^ Although recent studies showed promising clinical results for osteopathic medicine in the context of prematurity, a recent review of the published literature on pediatrics has demonstrated elusive results.^[22]^ Despite the arguable methodology used in the review and the broader topic, the general quality of research included was considered poor.^[22]^ One understudied aspect regarding prematurity is the effect of OMT on LOS. No systematic reviews have been carried out considering this outcome. Therefore, a comprehensive review of evidence for this vulnerable population would be useful in understanding the emerging osteopathic literature and may help provide a target for future interventions within the existing NICU health care programs. The aim of the present systematic review was to assess the extent to which osteopathic medicine is effective compared to the control group in reducing LOS, hospital costs, and adverse events in premature infants.

## 2 Materials and methods

### 2.1 Studies

The current systematic review included single- and multicenter randomized controlled trials (RCTs), quasi-RCTs, and controlled clinical trials. Due to the lack of controlled studies on the topic we included controlled before and after studies, with at least 1 intervention and 1 control site; interrupted time series studies which present at least 2 points of outcome measurement pre- and postintervention. Studies which have LOS as the primary outcome, or those which reported LOS as the secondary outcome to a health care program, were included. No language restriction was applied. Trials which were solely concerned with the collection of data from infants were excluded. In addition, case-control, case-series, case-report, conference proceedings, and abstracts were excluded.

### 2.2 Population

The eligible population for this review was preterm infants clinically stable and those recuperating from acute illness.

### 2.3 Intervention

The intervention of interest is OMT. The term OMT currently includes nearly 25 types of manual manipulative treatment technique. These techniques are used to treat SD within the body's framework, including skeletal, arthrodial, and myofascial structures.^[11]^ OMT procedures have been classified as direct or indirect.^[11]^

Eligible studies had to treat preterm infants directly in the crib or bed and OMT had to be performed by osteopaths. Due to the intrinsic clinical variability of manual techniques in terms of magnitude, frequency and time, no dosage restrictions (frequency and time) were applied. The comparisons were either sham therapy or no treatment. Studies including combined manual treatments were excluded. The OMT intervention and/or sham treatment could be administered in combination with usual/routine care.

### 2.4 Outcomes

The primary outcome was the mean difference in LOS measured in days between the osteopathy group and the alternative treatment group.

Secondary outcomes were:

- cost reduction (measured in Euros);
- weight gain, average weight in grams per kilo per day, considered as either continuous (mean difference) or categorical variable (categorized by the Z score);
- morbidity, measured as adverse clinical events, that is respiratory, gastrointestinal, neurological, cardiovascular, and genitourinary side effects (measured as the proportion of subjects with clinical complications in the osteopathy group compared to comparisons);
- long-term neuro-developmental outcomes measured at 1, 3, 6, 12, and 24 months using motor and cognitive tests.

### 2.5 Search methods

The identification of the studies was conducted by a comprehensive computerized search of Science Direct, MEDLINE, SCOPUS, Scholar Google, clinicaltrial.gov, the Cochrane Library, chiloras/MANTIS, Pubmed Europe, OSTMED.DR, and Osteopathic Research Web. Other sources considered were as follows: web searching, grey literature, conference proceedings, national trials registers. Terms used for the search were summarized as follows: osteopathic (MeSH and Free term), manipulat∗ (Free term and MeSH), treatment (Free Term and MeSH), medicine (MeSH), premature∗ (Free term and MeSH), infant∗(Free term and MeSH), preemie∗(MeSH), and newborn∗(MeSH).

The research was conducted from journal inception to May 17, 2015. Duplicate records were identified in EndNOTE and eliminated.

### 2.6 Selection of study

Two reviewers (DL and NR) conducted study selection independently based on the explicit search strategy. Discrepancies were resolved by consensus with an external arbiter. According to inclusion criteria, reviewers independently screened titles and abstracts. Full-texts were then retrieved and assessed. An independent pediatrician (GL) and an osteopath (MA) controlled the scientific relevance of the literature.

### 2.7 Data collection and evaluation

Two reviewers, considering type of interventions, number of patients, study results, and other descriptive characteristics of the included trials, carried out data extraction independently. Disagreements were discussed and reported by consensus. If data were not reported in the study, the authors were contacted. All data were kept on a specific hard disk, managed only by the 2 reviewers.

Each study was assessed according to the Cochrane risk of bias methods. A 4-point Likert scale was used for evaluation and included: very low, low, high and unclear risk of bias scores across 5 domains: sequence generation; allocation concealment; blinding to personnel; blinding to outcome analysis and other bias.^[23]^ CBA and ITS studies were evaluated using the tools proposed by the Cochrane Effective Practice and Organization of Care Group.^[24]^

### 2.8 Measures of treatment effect

Continuous data were analyzed using mean differences with 95% confidence intervals (MD; 95% CI). Dichotomous outcomes were presented as relative risk (RR) with 95% confidence intervals (CI). Missing data were handled by contacting the authors for more information. Where missing data were known, the reasons were described.

### 2.9 Assessment of heterogeneity

Studies were pooled only if there was significant homogeneity. This was assessed using the *I*^2^ statistic, which assessed how much of the variation between studies is due to heterogeneity rather than to chance.^[25]^ Values over 75% suggested considerable heterogeneity, but its significance also depended upon the magnitude and direction of the effect and the strength of the evidence. Reasons for heterogeneities may be multiple including methodological, statistical or clinical heterogeneity. To identify possible publication bias, funnel plots were used.^[25]^

### 2.10 Data synthesis

An intention-to-treat analysis was conducted. Data were reported as mean, point estimate, percentage, and range. Dispersion was presented as standard deviation (SD) and 95% confidence interval (CI). In the case of dichotomous outcome, the relative risk (RR) was greater than 1 if more patients were successfully treated by the osteopathy group than by the intervention group. An estimated pooled weighted average of RRs, using the Mantel–Haenszel fixed-effect method, with a 95% CI, was calculated. Due to the possibility that same studies reported continuous data as median and range, methods in the estimation of the sample mean and standard deviation were used.^[26]^

Where meta-analysis was not possible, results were presented using summary and descriptive statistics.

When feasible, subgroup analyses were focused on: age at birth (categorizing in less than 31 weeks, between 32 and 34 weeks, and between 35 and 37 weeks), time to first osteopathic session, diagnosis-related group and type of treatment. Review Manager v. 5.2.6 and R statistical software v3.12, packages “meta” were used for statistical analysis.

## 3 Results

**Figure 1:**
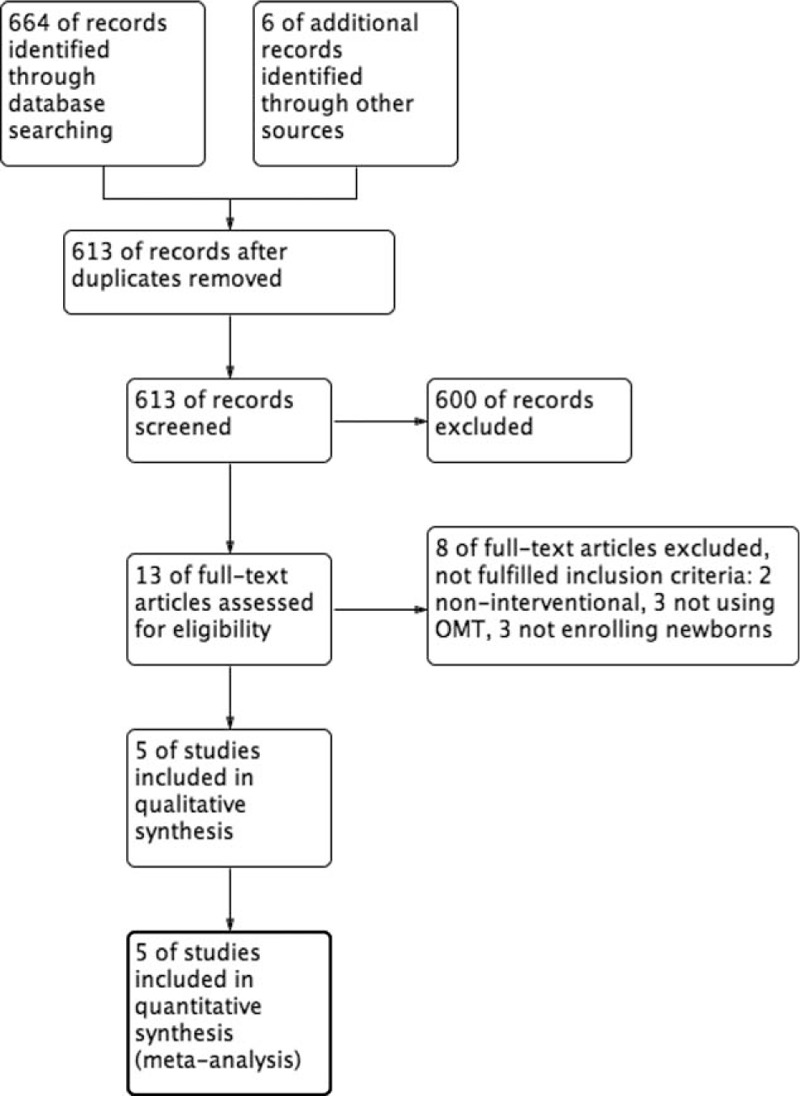
Flow diagram showing selection of articles.

The review process is shown in the flowchart of the study (Fig. 1): 670 articles and abstracts were identified from the initial searches. After removal of duplicates, 613 articles were assessed for eligibility; 600 were excluded, as it was clear from their abstracts or titles that they did not meet the inclusion criteria. Common reasons for rejection included the use of techniques and approaches different from OMT, focus on a different pediatric population, the use of noninterventional trials and reviews on general pediatric sample. The remaining 13 were screened for full-text review, of which 8 were excluded for the reasons showed in Fig. 1. Five studies met our inclusion criteria and were finally included in the systematic review.

### 3.1 Characteristics of the included studies

The included studies had a total population number of 1306 preterm infants born in European public NICUs. Four studies were conducted in Italy^[27–30]^ and 1 in Austria.^[31]^ The publication period ranged from 2007 to 2015.

Methodology used across studies revealed high homogeneity in terms of method of recruitment, recruitment setting, population, and type of control group.

All studies considered LOS as clinical outcome, 4 trials as primary outcome,^[27–30]^ whereas 1 considered it as a secondary outcome.^[31]^ Further studies’ aims were: average daily gut symptoms,^[27]^ costs,^[28–30]^ daily weight gain,^[28–30]^ meconium evacuation, full enteral feeding, and weight gain at discharge home.^[31]^

A fairly homogeneous study design was revealed, 4 were RCTs^[28–31]^ and 1 a nonrandomized observational study.^[27]^ Four out of 5 trials were single-center^[27–29,31]^ and 1 multicenter.^[30]^ Newborns population ranged from severe to late prematurity (Table 1).

**Table 1:**
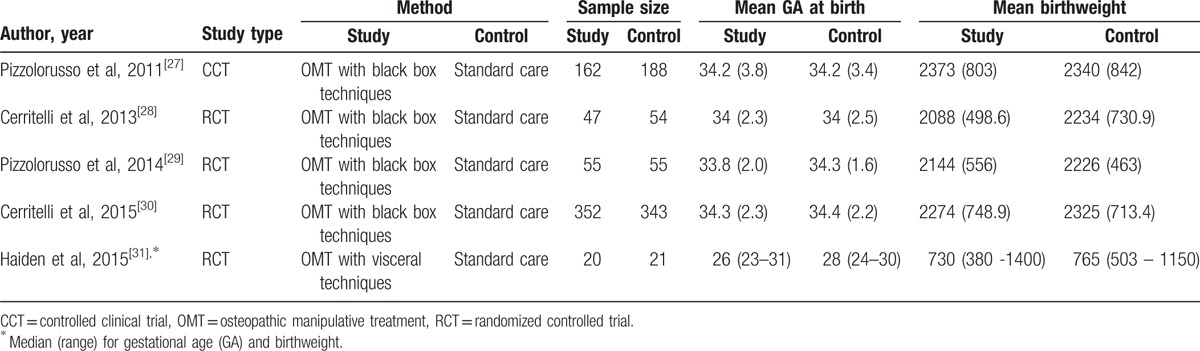
Characteristics of studies.

### 3.2 Intervention details

Four out of 5 studies administered a need-based approach,^[27–30]^ whereas Haiden et al^[31]^ used a pre-determined structured treatment protocol. Techniques used were indirect^[27–30]^ and visceral.^[31]^ Specific indirect techniques were: myofascial release, balanced ligamentous/membranous tension, indirect fluidic, and v-spread. The duration of treatment protocol was reported in 4 trials only^[27–30]^ and ranged from 20 to 30 minutes. Frequency varied from 2 to 3 times per week and the treatment period was for either the entire hospitalization^[27–30]^ or 1 week.^[31]^ The only study, which reported details on the type and sequence of intervention, and the treatment plan was Haiden et al.^[31]^

In all studies, treatment was performed either by osteopaths^[27–30]^ or by student in osteopathy,^[31]^ but only 2 studies^[28,29]^ gave data on the number of practitioners enrolled, 4 and 2, respectively.

Control groups used were standard care protocols. In addition to usual care, 3 studies^[28–30]^ included osteopathic evaluation, without any treatment, which mimicked the OMT session in terms of duration, dose, and length. All osteopathic interventions and control procedures were administered in the hospital.

### 3.3 Study quality-risk of bias

#### 3.3.1 Allocation

Low risk of sequence generation bias was scored for all studies, which employed an adequate randomization method, except for Pizzolorusso et al.^[27]^ as a non-RCT study design. Due to the nature of the population, allocation concealment was assessed as low risk of bias.

#### 3.3.2 Blinding of personnel and outcome assessors

Three studies reported that NICU staff was blinded to patient allocations and also unaware of study design and outcomes. One study had a high risk of bias as osteopathic physicians and patients were unblinded,^[31]^ whereas another trial did not report data on blinding of participants and personnel producing an unclear risk of performing bias.^[27]^

#### 3.3.3 Selective bias

Study protocols were made available for all research except for Pizzolorusso et al.^[27]^ Low risk of attrition and reporting bias were thus reported for 4 studies,^[28–31]^ whereas unclear for 1 study only.^[27]^

#### 3.3.4 Other bias

**Figure 2:**
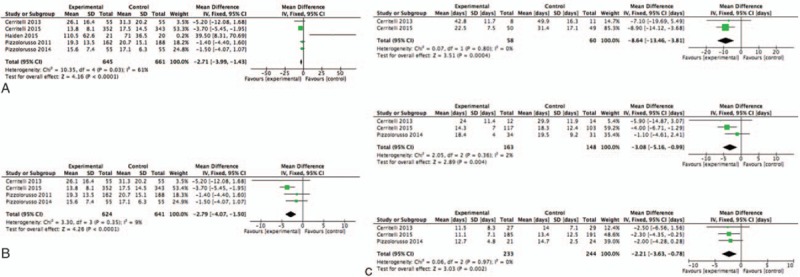
Summary of risk of bias for included studies.

The quality of studies included was further assessed considering: conflict of interest, funding source, ethical approval, informed consent, confidentiality, declaration of interests, access to data, trial registration, data collection, data management, and data monitoring committee. All studies included, apart from Pizzolorusso et al,^[27]^ reported sufficient and appropriate information on source of funding, ethical statement, and informed consent approval. Trial registration details were also reported. Regarding data collection, conflict and declaration of interest, trials included reported adequate information. None of the research detailed any information regarding confidentiality, access to data, data management, and data monitoring committee (Fig. 2).

### 3.4 Primary outcome: length of hospital stay (LOS)

#### 3.4.1 OMT vs usual care

**Figure 3:**
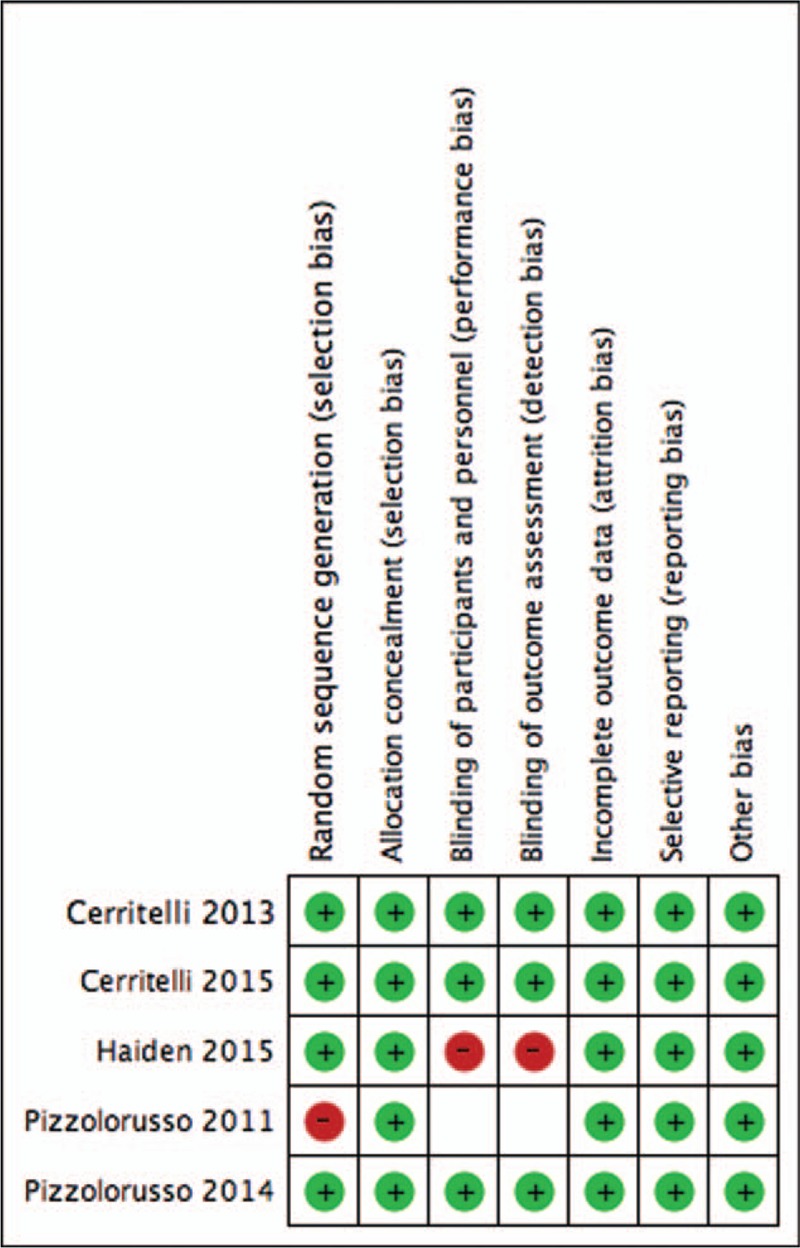
(right) Forest plot showing meta-analysis of osteopathic intervention on LOS. (A) comprehensive analysis including all studies. (B) Sensitivity analysis. (left) Sensitivity analysis by gestational age (GA). (A) Very preterm infant with GA < 32 w; (B) moderate preterm infants with 32 > GA < 34 w; (C) late preterm infants with GA >34 w. LOS = length of stay, GA = gestational age.

All 5 studies assessed the LOS (n = 1306, 645 preterm were allocated in the OMT group and 661 to the control group). Although the heterogeneity was moderate (*I*^2^ = 61%, *P* = 0.03), meta-analysis of all 5 studies showed that preterms who received OMT in addition to usual care had a significant reduction of LOS by 2.71 days (95% CI –3.99, –1.43; *P* < 0.001, Fig. 3) compared to those who did not undergo any osteopathic care.

### 3.5 Secondary outcomes

#### 3.5.1 Cost per infant per hospitalization

Three of the 5 studies included contained data for costs (n = 915, 462 preterm infants treated with OMT and 453 as control). Meta-analysis of these 3 studies showed that preterm infants had a significant lower costs compared to those allocated in the control group (–1545.66€, –1888.03€, –1203.29€, *P* < 0.0001, Table 2), although results indicated high heterogeneity (*I*^2^ = 90%, *P* < 0.0001).

**Table 2:**
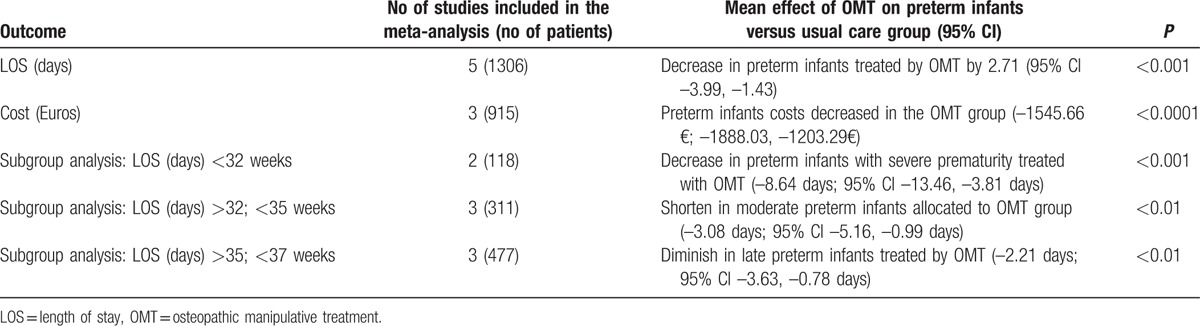
Summary of results of meta-analysis.

#### 3.5.2 Morbidity

All studies reported no adverse events associated to osteopathic intervention. Arguably, Haiden et al^[31]^ claimed in their study that the longer time to full enteral feeding in the OMT group could be interpreted as a possible adverse effect.

Cerritelli et al^[27,30]^ were the only 2 studies reporting drop-out data. In the 2015 study, authors showed that the rate in the OMT-study group was 2.2% (8/360) versus 4.7% (17/360) in the usual care control group (*X*^2^ = 3.36; RR = 0.47; 95% CI 0.21–1.08; Z = 1.76, *P* = 0.07), whereas in the 2013 trial, authors described that 8 preterm infants were lost during the trial compared to 1 on the control group.

#### 3.5.3 Long-term outcomes

None of the included studies measured long-term neuro-developmental outcomes at any time-point and using any neuro-developmental assessments.

### 3.6 Subgroup analyses

Subgroup analyses were carried out according to gestational age, considering severe (<32 weeks), moderate (32.0–33.6 weeks), and late (34.0–36.6 weeks) prematurity (Table 2).^[32]^

#### 3.6.1 Very preterm infants

Two studies enrolled newborns born before the 32nd week of gestation (n = 118, 58 received OMT and 60 were allocated to the control group). Meta-analysis showed that preterm infants who underwent osteopathic care were discharged earlier compared to controls by a mean of approximately 9 days (95% CI –13.46, –3.81 days; *P* < 0.001; see Fig. 3).

#### 3.6.2 Moderate preterm infants

Meta-analysis of the 3 studies that had comparative characteristics for reporting data on moderate prematurity (n = 311, with 163 treated with OMT and 148 controls) showed that preterm infants allocated to the osteopathy group were discharged significantly earlier than preterms in the control group by a mean of 3.08 days (95% CI –5.16, –0.99 days; *P* < 0.01; see Fig. 3).

#### 3.6.3 Late-preterm infants

Late preterm infants were considered in 3 studies (n = 477, with 233 in the OMT group and 244 in the control group). Meta-analysis showed that infants receiving OMT were discharged significantly earlier compared to the control group by a mean of more than 2 days (95% CI –3.63, –0.78 days; *P* < 0.01; see Fig. 3).

## 4 Discussion

The present systematic review aimed at evaluating the effectiveness of OMT on preterm infants. It included 5 RCTs involving 1306 patients. Analysis of the studies available for this review suggested that the administration of osteopathic medicine to infants born prematurely produced a significant reduction of LOS by almost 3 days on average. This will lead to a reduction of costs for the local health care system of more than €1500 per preterm per LOS.

Interestingly, the earlier the OMT intervention, the larger the benefit for newborns. In fact, it can be highlighted as high prematurity newborns (<32 wk GA) can benefit of the highest osteopathic effect (reduction of almost 9 days), whereas late preterm infants of the lowest (2 days). These results can be better understood in light of the differences among the 3 preterm subgroups (GA < 32; 32–33.6; 34–36.6 wks): average duration of LOS, longer for very preterm infants; policy of local hospitals (likelihood of admitting a late preterm is significant lower), and need for cure (high premature infants require longer, and more accurate cares). As far as adverse events were concerned, none of the trials included reported any adverse event. This could imply that osteopathic treatment could be considered a safe procedure.

Considering the characteristics of trials: included studies were conducted in clinical settings and in different European countries, although 4 out of 5 studies were carried out in Italy, suggesting that the findings would be best applicable in those contexts. Research included treatments administered by experienced practitioners with no apparent impact on outcomes. It should be pointed out that the studies were homogeneous in design and study outcomes, therefore suggesting reliability and validity of results.

### 4.1 Implications for practice

OMT seems to be effective in the treatment of premature babies in terms of reducing days of hospitalization and costs (at the net of osteopathic costs). Moreover, it was shown to be a safe procedure, considering short-term outcomes.

This review introduces new evidence that the use of osteopathy in very preterm infants produces greater improvements compared to late ones. From a clinical standpoint, the use of osteopathic procedures would seem to be a desirable choice when compared to usual care only. As a possible impact on local healthcare system, the use of osteopathy for preterm infants might be recommended as adjuvant therapy within the NICU routine practice. This could support the idea of integrated multidisciplinary medicine, promoted from the WHO and be in line with the aim of local and central healthcare strategies on costs’ optimization. From a policy perspective, potential implications can be predicted in terms of: health care system implementation, accessibility to complementary care and sustainability of health care costs. However, the implementation of healthcare system needs to be considered in light of: (a) robust results: several aspects of osteopathic research are still missing, that is, specific disease-based effects, elective care vs acute care; (b) evidence-based multidisciplinary practice: the role of osteopathy within routine-based multidisciplinary NICU environment; (c) evolution of neonatology care: deep understanding of current processes delivering healthcare procedures in neonatology.

### 4.2 Possible mechanisms of action

It has been suggested that preterm infants are associated with higher levels of pro-inflammatory circulating substances and immaturity of the vegetative system. Matoba et al^[33]^ reported more recently that levels of 12 biomarkers (IL-2, IL-4, IL-5, IL-8, IL-10, MCP-1, MIP-1a, MIP-1b, sIL-6ra, sTNF-RI, TNFα and TREM-1) were higher in preterm compared to term infants, whereas IL-1b and IL-18 were lower. Another recent study by McElrath et al^[34]^ observed that cytokines measured on the first day of life were higher in preterm infants born after complications associated with infections, compared to preterm infants born after complications like pre-eclampsia. Furthermore, preterm infants appear to have an abnormal maturation of autonomic nervous system. Yiallourou et al^[35]^ reported that compared to term infants, preterms exhibit a diminished parasympathetic modulation of the heart associated to greater respiratory-mediated changes and lower sympathetic modulation of blood pressure. In addition, Longin et al^[36]^ demonstrated that gestational age of newborns is correlated with a change in the heart rate variability.

Speculating on the possible mechanisms of action of osteopathy on preterm infants, it can be argued that OMT seems to be associated with a reduction of pro-inflammatory substances both in vitro and in vivo ^[37,38]^ hypothesizing an anti-inflammatory role of OMT, although only partially confirmed by recent clinical-based research.^[39]^ Therefore, OMT could reduce the release of cytokines and the sympathetic activity creating a cascade of biological and neurological events able to modulate both inflammatory and ANS mechanisms.^[40,41]^

More recently, preliminary lab-based evidence showed the effect of specific osteopathic techniques on the enhancement of the lymphatic and immune system ^[42,43]^ by improving the leukocytes count and interleukin-8 (IL-8). Findings were confirmed by a research article published in 2014 where significant differences were detected in the levels of immune molecules, including IL-8, between OMT and sham light-touch control.^[44]^ OMT, therefore, could have an effect on the immunological profile of specific circulating cytokines and leukocytes. Nevertheless, the lack of specific studies on newborns prevents any formal assumption on the biological effects of osteopathy on this population, calling, therefore, for more systematic and pragmatic trials.

### 4.3 Limitations

This review followed the recommendations of the Cochrane Collaboration, using sensitivity analysis as necessary. The method used for data analysis used assumptions that could not be verified because there was no access to the original data. Specifically, it was assumed that data had a parametric distribution; this may not be correct in particular for costs. Thus, this may have yielded conservative results, that is, underestimation of differences. Despite the total number of patients, the subgroup analyses showed small sample sizes. This could be considered critical for outcome variables (e.g., LOS). A further limitation is relative to the medication plan, of which reporting was lacking in all studies. It can be argued that drugs’ effects can significantly influence the clinical benefit of treatment. In addition, screening the papers’ authorships it should be noticed that more authors were involved in 4 out of 5 included papers. This could potentially lead to bias. However, the likelihood of a significant influence on the validity of the results is very low, due to the large sample size included in the review. Finally, another limitation to the consideration of osteopathic procedure as necessary within the management of preterm infants, especially when very premature, consists in the lack of data showing the effect of OMT on long-term respiratory and neurological outcomes.

## 5 Conclusion

The present systematic review showed the clinical effectiveness of OMT on the reduction of LOS, with subsequent economic advantages, in a large population of preterm infants. Despite the positive results, further research is needed to define potential limitations on the type of patients for whom osteopathic medicine is appropriate and to determine the optimal clinical and research scenario at which to intervene with osteopathic treatment. Moreover, such trials should aim at monitoring and reporting adverse events as well as long-term growth and neurodevelopmental outcomes, whereas addressing the possibility that nutritional intake, drug administration and NICU empowerment strategies might mask the effects of osteopathic treatment. Furthermore, upcoming research should study the underpinning mechanisms of osteopathic treatment in preterms, both healthy and with specific pathologies, to allow further advances in the biological understanding of manual medicine effects. This can be predicted as a milestone to plan future clinical neonatological applications of osteopathic medicine. In conclusion, based on the previous literature, osteopathy may offer further advantage over routine care only.

## Acknowledgements

The authors are grateful to Dr. James Fontini and Dr. Jorge Esteves for their kind comments during proofreading, and Luca Cicchitti for his contributions to the study.
